# Bacteriophage Application for Difficult-To-Treat Musculoskeletal Infections: Development of a Standardized Multidisciplinary Treatment Protocol

**DOI:** 10.3390/v11100891

**Published:** 2019-09-23

**Authors:** Jolien Onsea, Patrick Soentjens, Sarah Djebara, Maia Merabishvili, Melissa Depypere, Isabel Spriet, Paul De Munter, Yves Debaveye, Stefaan Nijs, Paul Vanderschot, Jeroen Wagemans, Jean-Paul Pirnay, Rob Lavigne, Willem-Jan Metsemakers

**Affiliations:** 1 Department of Development and Regeneration, KU Leuven, 3000 Leuven, Belgium; stefaan.nijs@uzleuven.be (S.N.); paul.vanderschot@uzleuven.be (P.V.); willem-jan.metsemakers@uzleuven.be (W.-J.M.); 2 Department of Trauma Surgery, University Hospitals Leuven, 3000 Leuven, Belgium; 3 Centre for Infectious Diseases, Queen Astrid Military Hospital, 1120 Brussels, Belgium; psoentjens@itg.be (P.S.); sarah.djebara@mil.be (S.D.); 4 Department of Clinical Sciences, Institute of Tropical Medicine, 2000 Antwerp, Belgium; 5 Laboratory for Molecular and Cellular Technology, Queen Astrid Military Hospital, 1120 Brussels, Belgium; maia.merabishvili@mil.be (M.M.); jean-paul.pirnay@mil.be (J.-P.P.); 6 Department of Laboratory Medicine, University Hospitals Leuven, 3000 Leuven, Belgium; melissa.depypere@uzleuven.be; 7 Pharmacy Department, University Hospitals Leuven, 3000 Leuven, Belgium; isabel.spriet@uzleuven.be; 8 Department of General Internal Medicine, University Hospitals Leuven, 3000 Leuven, Belgium; paul.demunter@uzleuven.be; 9 Department of Microbiology, Immunology and Transplantation, KU Leuven, 3000 Leuven, Belgium; 10 Department of Intensive Care Medicine, University Hospitals Leuven, 3000 Leuven, Belgium; yves.debaveye@uzleuven.be; 11 Laboratory of Gene Technology, KU Leuven, 3001 Leuven, Belgium; jeroen.wagemans@kuleuven.be (J.W.); rob.lavigne@kuleuven.be (R.L.)

**Keywords:** bacteriophage therapy, antibiotic resistance, multidisciplinary team, musculoskeletal infection

## Abstract

Bacteriophage therapy has recently attracted increased interest, particularly in difficult-to-treat infections. Although it is not a novel concept, standardized treatment guidelines are currently lacking. We present the first steps towards the establishment of a “multidisciplinary phage task force” (MPTF) and a standardized treatment pathway, based on our experience of four patients with severe musculoskeletal infections. After review of their medical history and current clinical status, a multidisciplinary team found four patients with musculoskeletal infections eligible for bacteriophage therapy within the scope of Article 37 of the Declaration of Helsinki. Treatment protocols were set up in collaboration with phage scientists and specialists. Based on the isolated pathogens, phage cocktails were selected and applied intraoperatively. A draining system allowed postoperative administration for a maximum of 10 days, 3 times per day. All patients received concomitant antibiotics and their clinical status was followed daily during phage therapy. No severe side-effects related to the phage application protocol were noted. After a single course of phage therapy with concomitant antibiotics, no recurrence of infection with the causative strains occurred, with follow-up periods ranging from 8 to 16 months. This study presents the successful outcome of bacteriophage therapy using a standardized treatment pathway for patients with severe musculoskeletal infection. A multidisciplinary team approach in the form of an MPTF is paramount in this process.

## 1. Introduction

Despite infection prevention measures, infectious complications after the implantation of orthopedic devices, such as fracture fixation materials or prostheses, still impose a heavy burden on patients and healthcare systems [1,2]. Treatment is challenging, owing to an increase in bacterial resistance to commonly used antibiotics, and the potential of bacteria to become attached to the surface of the implant and organize themselves into biofilms. Bacteria embedded in biofilms have a high survival and persistence potential because they are protected from environmental, chemical, and mechanical stresses [3]. Alternative antimicrobial strategies are therefore eagerly anticipated. One such strategy is bacteriophage therapy. Bacteriophages (phages) are viruses that are considered to be natural enemies of their host bacteria. Strictly lytic phages have three important properties that could make their therapeutic application successful. First, after they have infected their host bacterium, phages can self-amplify and infect other bacteria, which distinguishes them from conventional antimicrobials [4]. Second, some phages carry polysaccharide depolymerases, which improve the efficacy of phage infection by degrading the extracellular matrix of biofilm-associated bacteria [5]. Third, phages are considered to be safe because eukaryotic cells and human bacterial flora are not negatively affected [4,6,7].

The use of phages for the treatment of bacterial infections is not a novel concept, and has been applied since the start of the 20th century. However, with the advent of antibiotics, phage therapy lost ground in the Western world, while research and development continued to thrive within the former Soviet Union and Eastern Europe [8]. Given the current climate of antibiotic resistance, there is renewed interest in the application of phages [9], as illustrated by an increasing number of papers on this topic [10,11,12,13,14,15,16,17]. One of the biggest hurdles to implement phage therapy in the European Union (EU) is the lack of an appropriate legal and regulatory framework [10]. Phage therapy can only be applied to patients for whom all standard treatment options have been exhausted or are unavailable, i.e., in case of an “unmet medical need” conforming to Article 37 (i.e., unproven interventions in clinical practice) of the World Medical Association (WMA) Declaration of Helsinki (Fortaleza, Brazil, 2013). In Belgium, these regulatory hurdles have recently been overcome by the incorporation of phage therapy in the regulatory framework of magistral drug preparations [18]. If treating physicians consider that patients would likely benefit from phage therapy, the “magistral phage” framework allows the prescription of a customized phage cocktail.

Although phage therapy requires a very specific and customized treatment approach, to date no standardized application protocols or guidelines have been developed. From this perspective, we herein present the first steps towards the establishment of a “multidisciplinary phage task force” (MPTF) and a standardized treatment pathway, based on our own experience with the application of phage therapy in four patients with severe musculoskeletal infections.

## 2. Materials and Methods

### 2.1. Patient Identification

A multidisciplinary team (MDT), consisting of musculoskeletal trauma surgeons, microbiologists, infectious disease specialists, clinical pharmacists, and plastic surgeons, found four patients with severe musculoskeletal infections (osteomyelitis) eligible for phage therapy, based on the criteria included in Article 37 of the WMA Declaration of Helsinki (Fortaleza, Brazil 2013). These patients had a poor prognosis (i.e., need for amputation) after multiple failed medical and surgical therapy regimens, even though these therapy regimens were found adequate by the MDT. To set up individual treatment plans, the MDT collaborated with phage scientists and specialists. All patients were informed about their clinical situation and treatment options. Informed consent was obtained from each patient. Furthermore, the hospital’s Ethical Committee and the Chief Medical Officer gave formal consent on a case-by-case basis.

### 2.2. Isolation of Pathogens and Susceptibility Testing

All patients underwent thorough debridement, during which multiple (i.e., more than five) deep tissue and/or implant specimens were taken. Bacterial species and their antibiotic susceptibility patterns were determined using matrix-assisted laser desorption/ionization—time-of-flight mass spectroscopy (MALDI-TOF-MS) equipment (Bruker Daltonics, Inc., Billerica, MA, USA) and the VITEK^®^ 2 system (bioMérieux Inc., Marcy-l’Étoile, France), respectively. All isolated *Staphylococcus* spp. and *Pseudomonas aeruginosa* strains were tested for susceptibility to BFC1, a phage cocktail produced by the Queen Astrid Military Hospital (QAMH) in Brussels since 2007, which contains phages against *Staphylococcus aureus* (ISP) and *P. aeruginosa* (PNM and 14-1) [19]. The genetic profile was determined and the strictly lytic nature of these phages was ensured by Sciensano, the federal research institute for public health in Belgium. Unfortunately, for one patient, the isolated *P. aeruginosa* strain was not stored for susceptibility testing. The phages present in the BFC1 cocktail were diluted in 0.9% saline to a titer of 10^7^ plaque-forming units (PFU) per mL.

Because of a lack of phages against *Enterococcus faecalis* in the QAMH’s phage library, the commercial preparation Pyo bacteriophage (Eliava Institute, Tbilisi, Georgia)—which contains phages against *Streptococcus* spp., *Staphylococcus* spp., *Proteus* spp., *Escherichia coli*, *P. aeruginosa*, and additional phages against *Enterococcus* spp. [20]—was ordered from the Eliava Institute and tested against the *E. faecalis* strain isolated from one patient. The exact composition of phages or the titer of each phage is unknown for this cocktail [20]. Pyo bacteriophage is delivered in 10-mL vials and should be applied undiluted. Susceptibility to the phages present in each cocktail was tested using the spot test and double agar overlay method, as previously described [21]. The efficiency of plating (EOP), which is defined as the ratio of the phage titer on the test strain to the titer on the production strain, was also determined.

### 2.3. Intraoperative Administration of Bacteriophages

After thorough debridement and irrigation, a draining system was placed within the medulla or in close contact with the infected bone to enable rinsing with the selected phage solution. Prior to rinsing with the phage solution, sodium bicarbonate (1.4%) was injected to create an alkaline environment. The rinsing volume was determined based on the intraoperative situation. In cases of large bone/soft tissue defects (i.e., “dead space”), 40 mL of the phage solution was used to rinse the infected site. In those patients who underwent soft tissue coverage during the same procedure, smaller rinsing volumes were used, ranging from 10 to 20 mL. A contact time of 10 min was allowed, during which the drain was closed off with a Kocher’s surgical clamp. Before wound closure, a gentamicin-impregnated collagen sponge soaked in phage solution was placed on the infected bone.

### 2.4. Postoperative Administration of Bacteriophages and Patient Follow-up

The nursing staff were trained to apply the phage solution 3 times per day for 7–10 days, following the same protocol described above, at the patient’s bedside. For each patient, a specific application protocol was prepared, detailing rinsing volumes, the phage cocktail, and the duration of phage therapy. Each patient’s clinical status was evaluated daily, and blood tests were performed before surgery, after surgery, on days 2, 4, 7, 10, 14, and 28, and further according to the standard of care. Serum samples were stored at −80 °C for the phage neutralization assay.

All patients received concomitant antibiotics based on the susceptibility profile of the isolated pathogens.

### 2.5. Phage Neutralization Assay

Phage neutralization by patient serum was performed according to Adams [22] with some modifications; 0.9 mL of each diluted sample (1:100) was mixed with 0.1 mL of phage/Pyo bacteriophage at a concentration of 10^7^ PFU/mL, and incubated at 37 °C for 30 min. The phages were then titered with host production strains to determine the number of non-neutralized phages. The clinical isolate from Patient 4 was used in the case of Pyo bacteriophage.

### 2.6. Sequence Analysis of Bacterial Isolates

The sequences of consecutively isolated strains from Patient 2 were analyzed. Genomic DNA from *S. epidermidis* strains 180411 and 181231 was extracted using a DNeasy UltraClean Microbial kit (Qiagen, Hilden, Germany) according to the manufacturer’s instructions. Sequencing libraries were prepared using Illumina Nextera DNA Flex, and sequenced on an Illumina MiniSeq sequencer (Illumina, Inc., San Diego, California, USA). Analysis of the sequencing data was performed on the Pathosystems Resource Integration Center (PATRIC) platform [23]. After quality control and trimming of the raw reads, the genomes were assembled *de novo* using SPAdes 3.8 software, and submitted to the National Center for Biotechnology Information (NCBI) for functional annotation in the Prokaryotic Genome Annotation Pipeline (PGAP) and publication in GenBank (accession numbers VANN00000000 and VANO00000000). The genomes were compared using progressiveMauve [24], and by performing single-nucleotide polymorphism (SNP) analysis using Snippy v4.3.8 software with *S. epidermidis* 180411 as the reference strain.

## 3. Results

Four cases with severe musculoskeletal infections were treated using phage and concomitant antibiotic therapy. The patients’ details are displayed in Table 1.

Three patients were treated for chronic osteomyelitis of the femur, and one for chronic osteomyelitis of the pelvis. Although previous surgical and antibiotic treatments were considered adequate and maximal by the MDT, all patients suffered from multiple relapses of the infection. The associated microbiology results are displayed in Table 2.

The results of susceptibility testing of the bacterial strains against phage cocktails are presented in Table 3. Phages from the applied cocktails were capable of propagating inside most of the susceptible strains. The EOP values ranged from 0.001 to 0.7. The isolated *Staphylococcus* spp. from patients 1, 2, and 3 were susceptible to BFC1. The *P. aeruginosa* strain from Patient 1 was not available for susceptibility testing. The extensively drug-resistant (XDR) *P. aeruginosa* in Patient 2 was non-susceptible to the phages (i.e., PNM, 14/1) present in BFC1. Given that the *S. epidermidis* strain was susceptible to BFC1, the MDT decided that it would be in the patient’s best interest to proceed with this cocktail. At the time of treatment of the first three cases, our center did not yet have access to the Pyo bacteriophage cocktail. This cocktail was ordered and tested against the isolated strain from Patient 4. Pyo bacteriophage was found to be active against the isolated *E. faecalis* strain. The EOP could not be determined because the applied cocktail (Pyo bacteriophage) was produced at the Eliava Institute, and we did not have access to the production strain. It was not possible to define how many phages were active against the clinical *E. faecalis* isolate because all the phage plaques on the tested strains had similar morphologies.

All patients received concomitant antibiotics covering all isolated pathogens (i.e., in case of polymicrobial infection) for 3 months, except for one patient (Patient 2) who received antibiotics for 6 weeks, after which an antibiotic-free interval of 2 weeks was respected before revision surgery for bone defect reconstruction.

**Figure 1:**
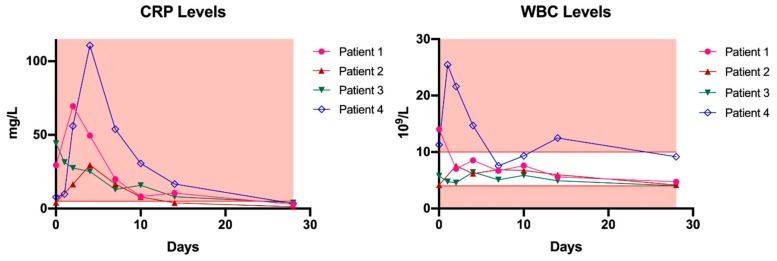
C-reactive protein (CRP) and white blood cell (WBC) levels in patients treated with phage therapy. The red background represents all values outside the reference range. Day 0 represents the baseline measurement, before phage therapy. Reference values: CRP: ≤5 mg/L, WBC: (4–10) × 10^9^/L.

Bacteriophage administration via the described route was generally well-tolerated, although one patient developed local redness and experienced pain during the rinsing procedure after seven days of treatment with the Pyo bacteriophage preparation. These symptoms were attributed to stowing, and subsided after phage therapy was stopped. However, a local immune reaction could not be ruled out because Pyo bacteriophage is not free from endotoxins. One month from the start of phage therapy, C-reactive protein (CRP) and white blood cell (WBC) levels returned to normal in all patients (Figure 1). Phage neutralization assays performed on these blood samples did not reveal any antibody production in any of the patients.

**Figure 2:**
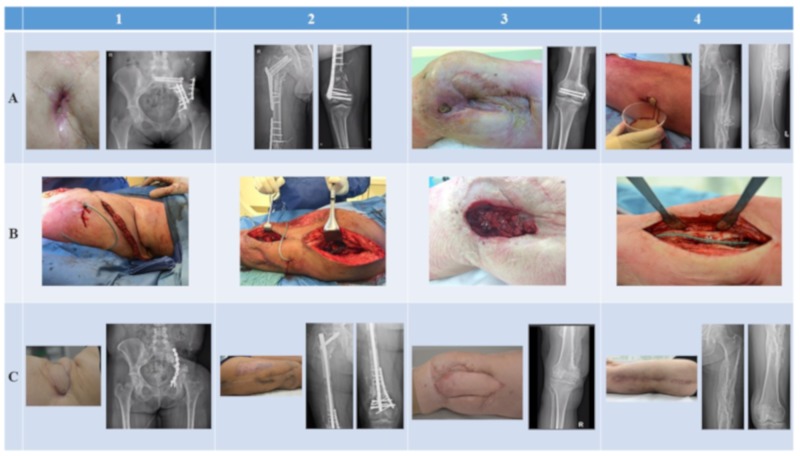
Clinical and radiological images of all patients treated with phage therapy. Each column represents a patient. Rows represent the preoperative (**A**), intraoperative (**B**), and postoperative (**C**) status of each patient.

Follow-up periods ranged from 8 months (Patients 3 and 4) to 16 months (Patients 1 and 2) after the start of phage therapy. In three patients (Patients 1, 3, and 4), clinical (i.e., inspection of the wound or scar, blood tests, general health status) and radiological investigations during routine outpatient visits showed no signs of recurrence (Figure 2C). These patients are currently infection-free. It was necessary for one patient (Patient 2) to undergo multiple complex surgical revision procedures for management of a bone defect, i.e., a bone transport procedure with long-term external fixation. Eight months after the initial treatment, an *S. epidermidis* strain (181231) was isolated again from multiple tissue samples during a bone grafting procedure at the docking site, although the antibiotic susceptibility pattern was distinct from that of the initial strain (180411). Full genome sequencing analysis was subsequently performed, and the presence of more than 8000 SNPs confirmed that these strains were not clonal. The XDR *P. aeruginosa* and the initial *S. epidermidis* strain could no longer be isolated during revision procedures in this patient. After several surgical revision procedures and antibiotic regimens, this patient is currently infection-free (Figure 2 and Figure 2C).

## 4. Discussion

Bacteriophage therapy for severe musculoskeletal infections has a number of historical precedents. In fact, in Eastern Europe, osteomyelitis is one of the major indications for which phage therapy has been applied during the last century, and patient reports have demonstrated high efficacy and safety [25,26,27,28]. However, it should be noted that these studies have important methodological limitations. In most studies, details of diagnostic criteria, phage production, phage cocktail composition, dosing, and the route of administration (e.g., topically, intravenously, orally) are missing. Furthermore, given the nature of phages and the need for customized cocktails that co-evolve with the host bacteria, phage cocktail production that conforms to Good Manufacturing Practice (GMP) guidelines remains a challenge [29]. This lack of solid clinical evidence has contributed to the fact that phage therapy is currently not officially registered as a legitimate treatment, and has often only been granted emergency approval for treatment failures [30].

Therefore, our primary goal was to standardize phage therapy in patients with severe musculoskeletal infections by introducing an MPTF. Overall, four patients were treated successfully and no recurrence of infection with any of the causative strains could be detected. At present, three of the four cases are considered infection-free—based on clinical, biochemical, and radiological evaluations—after surgical debridement in combination with phage therapy and concomitant antibiotics. One patient suffered a reinfection with a different strain from the initially isolated causative organisms after undergoing several revision surgeries for bone defect reconstruction. Furthermore, no severe systemic side effects or immune reactions were noted. In all patients, the systemic inflammatory markers (CRP and WBC count) decreased to normal levels after one month, and no antibodies were produced against the administered phages. The clinical data previously showed that the process of antibody production is highly phage-specific, and starts after 10 to 14 days of intravenous phage application [31]. In the present study, phages were administered to the patients for 7–10 days, and even though local administration through a draining system was cumbersome, the application method was generally well-tolerated.

This study has some limitations. The presented case results are preliminary and more cases are required to obtain high-quality evidence on the efficacy and safety of the presented phage therapy protocol in musculoskeletal infection. Secondly, the isolated strains from these first patients could only be tested against a limited panel of phages. The phages present in the BFC1 cocktail, which was applied in most cases, are well-characterized, in that the genetic sequence and therefore the confirmation of each phage’s strictly lytic profile and absence of undesired genetic determinants such as toxin and antibiotic resistance genes (i.e., “genetic passport”) are available. For this reason, this cocktail is preferred in our center. We currently do not have a large phage library available, and therefore our phage test panel is limited. Because of the medical history and clinical prognosis of the fourth patient, and after having gained experience with phage therapy in the first three patients, the commercial Pyo bacteriophage preparation was ordered. A limitation to the use of this cocktail is the fact that it is not purified from endotoxins and we do not know the exact composition of the cocktail. It is difficult to determine if the local reaction the patient developed after seven days of phage therapy is related to this, as it can also be attributed to stowing.

Nevertheless, our study stresses the importance of a multidisciplinary approach to the selection, treatment, and monitoring of patients receiving bacteriophage therapy. Moreover, this approach should lead to more standardized treatment pathways, which remains an important issue, as confirmed by recently published case reports [32,33,34,35]. Although these reports also describe a combination of phage therapy with antibiotics, they include a multitude of treatment schedules (e.g., routes of administration). Two case reports from the same group describe the successful combination of antibiotic and phage therapy in patients with chronic musculoskeletal infections refractory to multiple standard treatments. One patient with advanced lung cancer and a chronic fistula following radiation of a bone metastasis was treated every three days by the local application of a phage cocktail (against XDR *P. aeruginosa*). A contact time of four hours was allowed. The final titer of each bacteriophage in the cocktail was approximately 10^8^ PFU/mL [34]. Another case report presented a patient with a polymicrobial prosthetic joint infection (PJI) treated with phages against two pathogens (i.e., *P. aeruginosa* and *S. aureus*) by means of a single intraoperative injection of a phage cocktail. The titer that was eventually administered was not clear from this report [32]. Nir-Paz et al. describe the successful treatment of a fracture-related infection (FRI) caused by XDR *Acinetobacter baumannii* and multidrug-resistant (MDR) *Klebsiella pneumoniae* with phages and concomitant intravenous antibiotics. Phages were administered intravenously 3 times per day for 5 days. Subsequently, the *A. baumannii* strain was isolated again, after which a second intravenous course of phage therapy was started for an additional six days. The phage titer was not reported [33]. In a study that more closely resembles the present work, Vogt et al. described the eradication of infection in a patient with osteomyelitis of the femur, caused by a pan-resistant strain of *P. aeruginosa*. Pyo bacteriophage was administered several times per day via a draining system. The duration of phage treatment was not reported [35].

In Belgium, phage therapy was recently implemented in the legal framework for magistral preparations, which should allow the treatment of more infected cases [18]. However, the cases mentioned above and previously conducted clinical trials underline the fact that solid evidence on phage therapy efficacy and optimal administration protocols is currently lacking. With this in mind, the MPTF at our center will evaluate the optimal candidates that may benefit from phage therapy, and ensure adherence to a standardized treatment protocol. Patients are eligible when they are diagnosed with a severe musculoskeletal infection for which previous (surgical and antibiotic) treatments have failed. The MPTF consists of infectious disease specialists, pharmacists, microbiologists, surgeons, and phage scientists. They develop the treatment plan (i.e., concomitant antibiotics, surgeries, phage titer, dosage, duration of therapy) and follow-up with patients on a regular basis. The data collected during the procedures and follow-up visits will be stored in a prospective registry. The regular analysis of such a database will provide insight into the efficacy and safety of phage therapy in patients with a severe musculoskeletal infection.

## 5. Conclusions

In the present manuscript, we present successful outcomes in four patients with severe musculoskeletal infections after a single course of phage therapy combined with antibiotics. Although high-quality evidence for optimal treatment and application protocols is currently lacking, we showed that the application through a draining system is generally well-tolerated. Nevertheless, this procedure is cumbersome and the nursing staff require specific training. Carefully selecting and monitoring the patients that could benefit from phage therapy should provide insight into the value of phage therapy for the treatment of severe musculoskeletal infections and, consequently, enable the establishment of standardized treatment and application guidelines. A multidisciplinary approach in the form of an MPTF will be paramount in this process.

## Figures and Tables

**Table 1:** Patient characteristics.

	Patient 1	Patient 2	Patient 3	Patient 4
**Relevant medical history**	2013—Solitary fibrous tumor in left pelvic region. Treated with neoadjuvant radiotherapy and surgical resection; reconstruction with bone grafts, osteosynthesis and free muscle flap.	2017—Polytrauma after assault, with open segmental fractures of the right femur. Treated with debridements, staged open reductions, proximal and distal fracture fixation of the femur and synthetic bone grafting. Soft tissue coverage with a lateral Gastrocnemius flap.	1995—Polytrauma after building collapse, with crush lesions of the right upper leg, complex femur fractures, condylar fracture of the knee and compartment syndrome. Treated with plate fixation.	1981—Polytrauma after traffic accident, with femur fractures. Treated with fracture fixation.
**Infection onset and evolution**	Wound dehiscence and evolution to chronic osteomyelitis of the os ileum with a draining fistula	Non-union distal femur	Postoperative wound problems: multiple episodes of erysipelas, pus drainage, evolution into a draining fistula	Infection of the surgical site with abscess formation and eventually the evolution to osteomyelitis of the femur
**Diagnosis**	2015	2017	1995	1984
**Infected site**	Pelvis	Femur	Femur	Femur
**Isolated pathogen(s)**	*P. aeruginosa* ^a^ *S. epidermidis*	*P. aeruginosa* *S. epidermidis*	*S. agalactiae* *S. aureus*	*E. faecalis*
**Initial/previous treatments**	Multiple debridements Removal of the osteosynthesis material Hyperbaric oxygen therapy Multiple courses of antibiotic therapy Temporary coverage of the wound with negative-pressure wound therapy	Multiple debridements Removal of the osteosynthesis material Multiple courses of antibiotic therapy	Multiple debridements Removal of the osteosynthesis material Multiple courses of antibiotic therapy Local (topical) treatment	Multiple debridements Removal of the osteosynthesis material Placement of gentamicin-coated beads Multiple courses of antibiotic therapy Local (topical) treatment
**Antibiotic therapy used with phages and duration**	Vancomycin iv Rifampicin po ^b^ Moxifloxacin po ^c^ Total duration: 3 months	Vancomycin iv Colistin iv Fosfomycin iv Total duration: 6 weeks	Vancomycin iv ↔ Clindamycin po Moxifloxacin po Total duration: 3 months	Amoxicillin iv ↔ Amoxicillin po Total duration: 3 months
**Phages used and duration**	BFC 1 Total duration: 7 days	BFC 1 Total duration: 10 days	BFC 1 Total duration: 9 days	Pyo bacteriophage Total duration: 7 days

^a^ This bacterial strain was not stored; thus, phage susceptibility could not be tested. The pathogen was initially treated with intravenous antibiotics for a total duration of more than five months, after which the strain could no longer be isolated for several months. Definitive antibiotics used in combination with phages, therefore, do not cover this strain, but considering the patient’s medical history with multiple recurrences with the *P. aeruginosa* strain, even after previous treatment with colistin, it was decided to apply BFC 1. ^b^Rifampicin was started once wounds were dry and drains were removed. ^c^Antibiotic therapy was modified based on tissue culture results from the final surgery, which showed the presence of *Morganella morganii*, sensitive to fluoroquinolones. iv: intravenous therapy; po: oral therapy; ↔: switch from intravenous therapy to oral therapy.

**Table 2:** Isolated pathogens and associated antibiotic susceptibility profiles.

	AMX	TZP	CAZ	FEP	MEM	LVX	GEN	TOB	AMK	ERY	CLI	SXT	VAN	RIF	CST	OXA	LZD	MIN	PEN
**Patient 1**																			
*S. epidermidis*						R	R	R	S	R	R	R	S	S		R	S	S	
*P. aeruginosa*		R	R	R	R	R	I		S			R			S				
**Patient 2**																			
*S. epidermidis*						S	R	R	S	R	S	S	S	S		R	S	S	
*P. aeruginosa*		R	R	R	R	R	R	R	R			R			S				
**Patient 3**																			
*S. aureus*						R	S	S		S	S	S				S	S	R	
*S. agalactiae*										S	S								S
**Patient 4**																			
*E. faecalis*	S						R												

AMX: amoxicillin; TZP: piperacillin-tazobactam; CAZ: ceftazidime; FEP: cefepime; MEM: meropenem; LVX: levofloxacin; GEN: gentamicin; TOB: tobramycin; AMK: amikacin; ERY: erythromycin; CLI: clindamycin; SXT: trimethoprim-sulfamethoxazole; VAN: vancomycin; RIF: rifampicin; CST: colistin; OXA: oxacillin; LZD: linezolid; MIN: minocycline; PEN: penicillin; R: resistant strain; S: susceptible strain; I: susceptible strain, in case of increased exposure.

**Table 3:** Susceptibility testing of bacterial strains against phage cocktails. The bacterial strains from the first three patients were not tested against Pyo bacteriophage because this cocktail was not available in our center at the time. EOP, efficiency of plating; PFU, plaque forming units; NT, not tested; NA, not applicable; +, active; -, inactive.

Patient	Bacterial Strains	Activity of BFC 1	EOP of BFC 1	Activity of Pyo Bacteriophage	Titer of Pyo Bacteriophage PFU/mL
1	*S. epidermidis*	+	0.1	NT	NT
2	*P. aeruginosa* *S. epidermidis*	- +	NA 0.001	NT NT	NT NT
3	*S. aureus*	+	0.7	NT	NT
4	*E. faecalis*	NA	NA	+	1.00 × 10^7^

## References

[1] Metsemakers W.J., Kuehl R., Moriarty T.F., Richards R.G., Verhofstad M.H.J., Borens O., Kates S., Morgenstern M. (2018). Infection after fracture fixation: Current surgical and microbiological concepts. Injury.

[2] Trampuz A., Widmer A.F. (2006). Infections associated with orthopedic implants. Curr. Opin. Infect. Dis..

[3] Boudarel H., Mathias J.D., Blaysat B., Grediac M. (2018). Towards standardized mechanical characterization of microbial biofilms: Analysis and critical review. NPJ Biofilms Microb..

[4] Kutter E., De Vos D., Gvasalia G., Alavidze Z., Gogokhia L., Kuhl S., Abedon S.T. (2010). Phage therapy in clinical practice: Treatment of human infections. Curr. Pharm. Biotechnol..

[5] Pires D.P., Oliveira H., Melo L.D., Sillankorva S., Azeredo J. (2016). Bacteriophage-Encoded depolymerases: Their diversity and biotechnological applications. Appl. Microbiol. Biotechnol..

[6] Abedon S.T., Thomas-Abedon C. (2010). Phage therapy pharmacology. Curr. Pharm. Biotechnol..

[7] Curtright A.J., Abedon S.T. (2011). Phage therapy: Emergent property pharmacology. J. Bioanal. Biomed..

[8] Abedon S.T., Kuhl S.J., Blasdel B.G., Kutter E.M. (2011). Phage treatment of human infections. Bacteriophage.

[9] Moelling K., Broecker F., Willy C. (2018). A wake-up call: We need phage therapy now. Viruses.

[10] Furfaro L.L., Payne M.S., Chang B.J. (2018). Bacteriophage therapy: Clinical trials and regulatory hurdles. Front. Cell. Infect. Microbiol..

[11] Lin D.M., Koskella B., Lin H.C. (2017). Phage therapy: An alternative to antibiotics in the age of multi-drug resistance. World J. Gastrointest. Pharmacol. Ther..

[12] Akanda Z.Z., Taha M., Abdelbary H. (2018). Current review—The rise of bacteriophage as a unique therapeutic platform in treating peri-Prosthetic joint infections. J. Orthop. Res..

[13] Cisek A.A., Dabrowska I., Gregorczyk K.P., Wyzewski Z. (2017). Phage therapy in bacterial infections treatment: One hundred years after the discovery of bacteriophages. Curr. Microbiol..

[14] Azam A.H., Tanji Y. (2019). Peculiarities of staphylococcus aureus phages and their possible application in phage therapy. Appl. Microbiol. Biotechnol..

[15] Dufour N., Delattre R., Ricard J.D., Debarbieux L. (2017). The lysis of pathogenic escherichia coli by bacteriophages releases less endotoxin than by beta-Lactams. Clin. Infect. Dis..

[16] El Haddad L., Harb C.P., Gebara M.A., Stibich M.A., Chemaly R.F. (2019). A systematic and critical review of bacteriophage therapy against multidrug-Resistant eskape organisms in humans. Clin. Infect. Dis..

[17] Knoll B.M., Mylonakis E. (2014). Antibacterial bioagents based on principles of bacteriophage biology: An overview. Clin. Infect. Dis..

[18] Pirnay J.P., Verbeken G., Ceyssens P.J., Huys I., De Vos D., Ameloot C., Fauconnier A. (2018). The magistral phage. Viruses.

[19] Merabishvili M., Pirnay J.P., Verbeken G., Chanishvili N., Tediashvili M., Lashkhi N., Glonti T., Krylov V., Mast J., Van Parys L. (2009). Quality-Controlled small-Scale production of a well-Defined bacteriophage cocktail for use in human clinical trials. PLoS ONE.

[20] Villarroel J., Larsen M.V., Kilstrup M., Nielsen M. (2017). Metagenomic analysis of therapeutic pyo phage cocktails from 1997 to 2014. Viruses.

[21] Djebara S., Maussen C., De Vos D., Merabishvili M., Damanet B., Pang K.W., De Leenheer P., Strachinaru I., Soentjens P., Pirnay J.P. (2019). Processing phage therapy requests in a brussels military hospital: Lessons identified. Viruses.

[22] Adams M.H. (1959). Methods of study of bacterial viruses. Bacteriophages.

[23] Wattam A.R., Abraham D., Dalay O., Disz T.L., Driscoll T., Gabbard J.L., Gillespie J.J., Gough R., Hix D., Kenyon R. (2014). Patric, the bacterial bioinformatics database and analysis resource. Nucleic Acids Res..

[24] Darling A.E., Mau B., Perna N.T. (2010). Progressivemauve: Multiple genome alignment with gene gain, loss and rearrangement. PLoS ONE.

[25] (2014). Phage Therapy: Current Research and Applications.

[26] Chanishvili N. (2012). A Literature Review of the Practical Application of Bacteriophage Research.

[27] Slopek S., Weber-Dabrowska B., Dabrowski M., Kucharewicz-Krukowska A. (1987). Results of bacteriophage treatment of suppurative bacterial infections in the years 1981–1986. Arch. Immunol. Ther. Exp..

[28] Lang G., Kehr P., Mathevon H., Clavert J.M., Sejourne P., Pointu J. (1979). Bacteriophage therapy of septic complications of orthopaedic surgery (author’s transl]. Rev. Chir. Orthop. Repar. Appar. Mot..

[29] Jault P., Leclerc T., Jennes S., Pirnay J.P., Que Y.A., Resch G., Rousseau A.F., Ravat F., Carsin H., Le Floch R. (2019). Efficacy and tolerability of a cocktail of bacteriophages to treat burn wounds infected by pseudomonas aeruginosa (phagoburn): A randomised, controlled, double-Blind phase 1/2 trial. Lancet Infect. Dis..

[30] Patey O., McCallin S., Mazure H., Liddle M., Smithyman A., Dublanchet A. (2018). Clinical indications and compassionate use of phage therapy: Personal experience and literature review with a focus on osteoarticular infections. Viruses.

[31] Bearden C.M., Agarwal A., Book B.K., Vieira C.A., Sidner R.A., Ochs H.D., Young M., Pescovitz M.D. (2005). Rituximab inhibits the in vivo primary and secondary antibody response to a neoantigen, bacteriophage phix174. Am. J. Transplant..

[32] Ferry T., Leboucher G., Fevre C., Herry Y., Conrad A., Josse J., Batailler C., Chidiac C., Medina M., Lustig S. (2018). Salvage debridement, antibiotics and implant retention (“dair”) with local injection of a selected cocktail of bacteriophages: Is it an option for an elderly patient with relapsing staphylococcus aureus prosthetic-Joint infection?. Open Forum Infect. Dis..

[33] Nir-Paz R., Gelman D., Khouri A., Sisson B.M., Fackler J., Alkalay-Oren S., Khalifa L., Rimon A., Yerushalmy O., Bader R. (2019). Successful treatment of antibiotic resistant poly-Microbial bone infection with bacteriophages and antibiotics combination. Clin. Infect. Dis..

[34] Ferry T., Boucher F., Fevre C., Perpoint T., Chateau J., Petitjean C., Josse J., Chidiac C., L’Hostis G., Leboucher G. (2018). Innovations for the treatment of a complex bone and joint infection due to xdr pseudomonas aeruginosa including local application of a selected cocktail of bacteriophages. J. Antimicrob. Chemother..

[35] Vogt D., Sperling S., Tkhilaishvili T., Trampuz A., Pirnay J.P., Willy C. (2017). beyond antibiotic therapy—Future antiinfective strategies—Update 2017. Unfallchirurg.

